# Experimental study on failure mode and fracture evolution characteristics of red shale in Kaiyang Phosphorus mining area

**DOI:** 10.1038/s41598-024-60981-z

**Published:** 2024-05-03

**Authors:** Zhenqian Ma, Lang Zhou, Shaojie Zuo, Jimin Zhang

**Affiliations:** School of Mining, Guizhou University, Guiyang, 550025 Guizhou Province China

**Keywords:** Red shale, Bedding, PFC, Anisotropy, Displacement field, Solid Earth sciences, Engineering, Mathematics and computing

## Abstract

In order to study the failure mode and fracture evolution characteristics of red shale in Kaiyang Phosphorus mining area, conventional triaxial compression mechanical tests of red shale with different bedding dip angles were carried out by using DSTD-1000 electro-hydraulic servo rock mechanics experiment system. Based on the laboratory test results, the conventional triaxial particle flow simulation of red shale samples with different bedding dip angles was carried out using discrete element PFC2D. The results show that: (1) the failure mode of red shale is controlled by bedrock when the bedding dip angle is 0° and 60° ~ 90°. When the bedding dip angle is 15° ~ 45°, the rock failure mode is controlled by bedding. The compressive strength of rock is the minimum when the bedding dip angle is 30°and the maximum at 0°, which is about 2 times of the minimum. (2) In the failure process of red shale, the cracks with different bedding dip angles show slow growth stage, accelerated growth stage and stable stage with axial strain. The whole failure process is dominated by tensile cracks, accompanied by a few shear cracks. (3) The type of displacement field varies with the bedding dip angle: tensile failure and shear failure are the main displacement field types at 15° ~ 45°, and mixed failure is often the main mode at 60° ~ 90°and 0°. The research results provide the basis and reference for the safety control of red shale roadway.

## Introduction

Red shale is a typical joint rock mass, and the tendency and distribution of the joints play a decisive role in the macroscopic mechanical properties of rocks^1^, such as compressive strength, tensile strength, elastic modulus, making the rock mass show anisotropic characteristics, and has an important impact on the stability of underground engineering^2–4^. Therefore, further study on anisotropic mechanical properties and failure mechanism of red shale is the basis for safe construction and determination of reasonable support parameters of red shale roadway, which has important theoretical research significance and engineering value.

The red shale in Kaiyang phosphate mine area located in southwest China has obvious thin layer, and the layer spacing varies from a few millimeters to more than ten centimeters^5^. Under the influence of anisotropic mechanical properties, the strength and deformation characteristics of red shale are more complex. Wang^6^ reveals the instability mechanism of red shale roadway in the original rock stress environment or under mining disturbance by testing the composition, microstructure and strength characteristics of red shale. Li^7^ studied the changes of uniaxial tensile strength and cumulative energy of acoustic emission of red shale under different dry and wet conditions. Manchao^8^ and HADIZSDE^9^ have studied the variation of shale mechanical property parameters with water content by shale softening test. Li^10^, Taheri^11^ and Wang^12^ have carried out a large number of experimental and theoretical studies on the anisotropic mechanical behavior of bedding shale. However, it is difficult for macroscopic mechanical tests to reproduce the fracture evolution in rock, and can not accurately predict engineering disasters.

With the increasing development of numerical methods, some researchers have started numerical modeling of jointed rock sample. Taking joints as the research object, a large number of scholars at home and abroad have carried out in-depth research. Fatemeh^13^ investigate numerically the impact of joint angle, mechanical properties of joints, and joints spacing on the strength and failure mechanism of rock samples under unconfined and confined conditions. Yang^14^ simulated the mechanical properties of different bedding shales under triaxial compression by using the discrete element method (DEM), and obtained the variation of strength with bedding dip angle and confining pressure. Deng^15^ obtained the law of joint number and rock failure through the mechanical test of quasi-layered rock mass. Shang^16^, Lin^17^, Bahaaddini^18^ and Wang^19^ use the discrete element method to study the relationship between the failure mode and mechanical properties of rock mass with joints.

To summarize, previous studies have investigated the mechanical properties of bedding red shale, such as softening, compressive strength, and tensile strength. However, these studies didn’t quantitatively describe the relationship between fracture and bedding inclination when the specimen was damaged, changes in fabric before and after sample failure, or the evolution mechanism of particle displacement field driven by cracks. This paper established numerical models of red shale with different bedding layers using discrete element PFC2D, and the mesoscale parameters were calibrated based on the laboratory experiment results of red shale samples with 30° bedding Angle. From the aspects of fracture evolution process, the relationship between fracture and bedding inclination Angle, rock fabric changes before and after sample failure, and the evolution mechanism of particle displacement field driven by fracture, the changes of mesoscale failure characteristics with bedding inclination Angle are analyzed. The relationship between sample failure mode and bedding inclination Angle is discussed from a microscopic perspective. When the inclination is 15° ≤ θ ≤ 45°, most displacement field types are tensile failure (DT and RT) and shear failure (DS and RS). When the inclination Angle is 60° ≤ θ ≤ 90° and θ = 0°, the displacement field type is usually dominated by mixed failure modes (MF-1, MF-2 and MF-3).

## Rock mechanics test of red shale

### Sample preparation and experimental scheme

**Figure 1:**
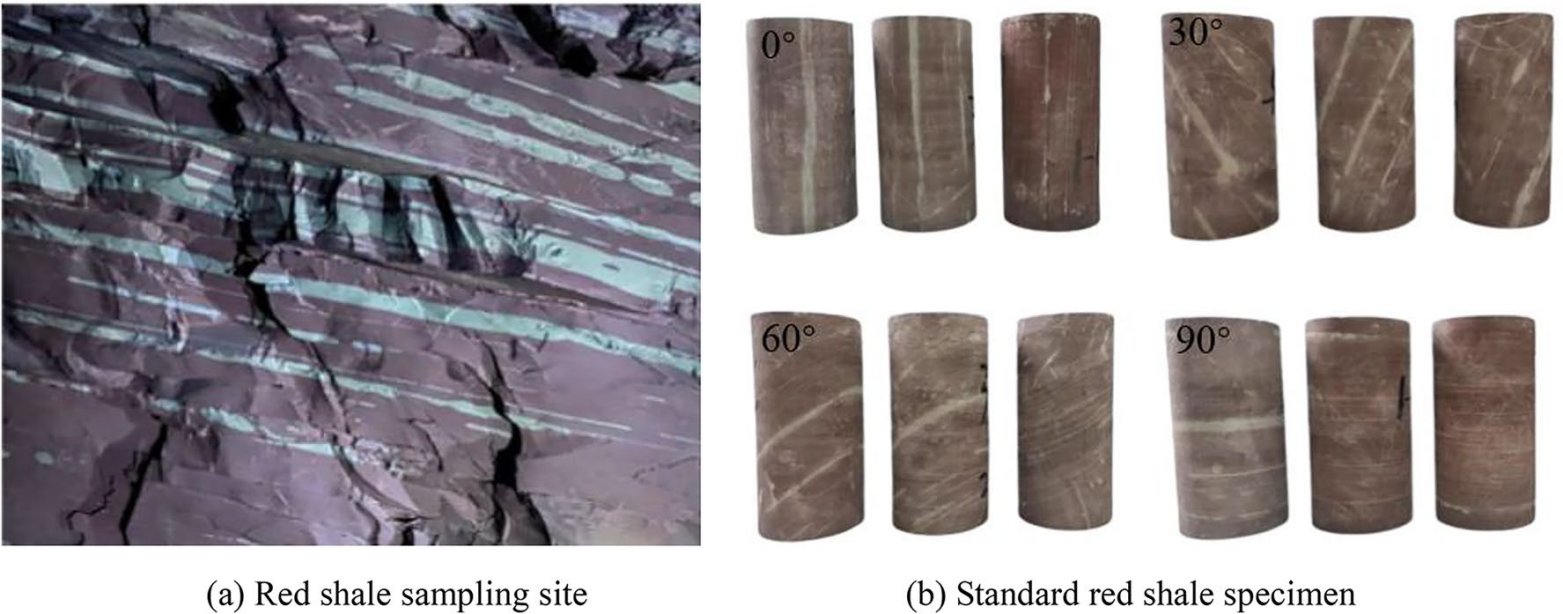
Red Shale samples preparation process.

The samples are taken from the red shale roadway buried in 600–700 m in the Kaiyang phosphate mining area in southwest China, and processed into Φ 50 mm × 100 mm cylindrical samples according to the standards of the International Society of Rock Mechanics, the unevenness error of the two ends of the specimen shall not be greater than 0.05mm^20^. A total of 36 samples were prepared, and the more discrete samples were removed by wave velocity detection, leaving 3 samples for each of the four kinds of bedding angles, as shown in Fig. 1. The DSTD -1000 electro-hydraulic servo rock mechanics test system was used in the experiment. The axial and radial strain of the samples were measured by high precision strain extensometer. Four sets of triaxial tests of red shale with bedding dip angles of 0°, 30°, 60° and 90° were designed. The displacement controlled loading method was adopted. The loading speed was 1 × 10^-4^ mm s^−1^ and the confining pressure was 2 MPa. In addition, the thickness of the bedding varies from 2 to 8 mm, mainly composed of clay minerals, while containing a large number of chlorite minerals, resulting in poor mechanical properties of the bedding surface.

### Analysis of test results

**Figure 2:**
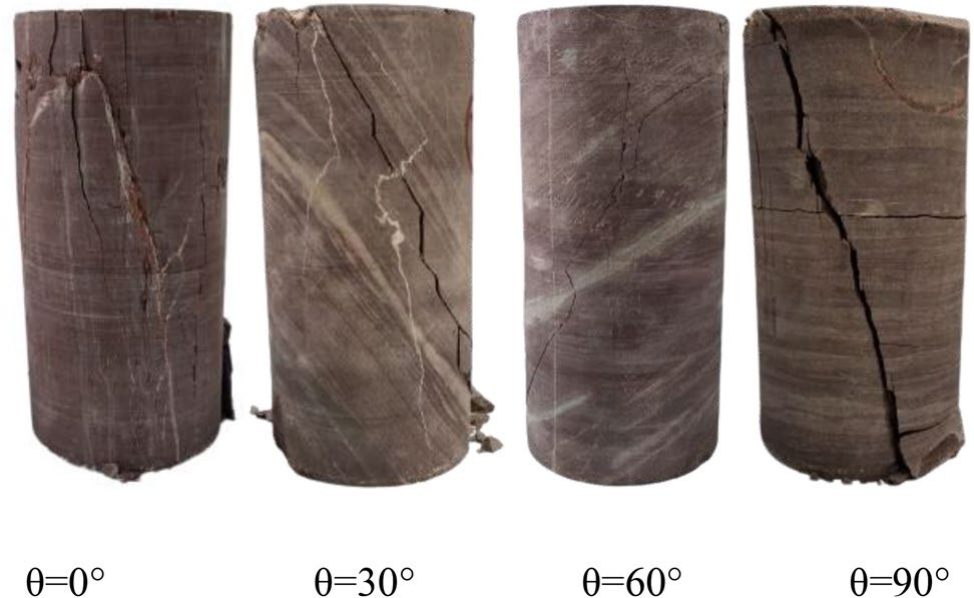
Diagram of crack propagation of specimen.

The test results are shown in Table 1. Figure 2 shows the failure modes of the specimens with different bedding angles. When the bedding dip angle is 0°, the specimen splits along the loading direction, the crack is parallel or sub-parallel to the loading direction, and the strength value is the maximum. When the bedding dip angle increases to 30°, the shear failure of the sample along the bedding position and runs through the whole sample, and the strength reaches 50.60 MPa. When the bedding dip angle is 60°, there is a tensile crack in the local position of the bedrock, which belongs to splitting failure, while shear failure occurs in the bedding. When the bedding inclination angle is 90°, the inclined crack runs through the specimen, which belongs to shear failure.

**Table 1:** Results of triaxial compression test.

Number	Bedding dip /°	Sample size /mm		*σ*_c_/MPa	*E*/GPa
		*D*	*H*		
1	0°	49.21	100.28	128.83	26.68
2		49.13	100.54	125.15	25.26
3		49.16	100.68	127.61	25.56
4	30°	49.10	99.75	51.86	25.36
5		49.25	100.16	49.41	23.95
6		49.29	100.12	50.52	23.52
7	60°	49.31	99.63	68.99	22.92
8		49.15	100.25	68.12	20.85
9		49.18	100.31	69.35	20.16
10	90°	49.31	99.12	105.36	16.45
11		49.12	100.58	104.62	16.09
12		49.32	100.12	103.85	15.23

The above analysis and test results show that the bedding is the weak surface of the specimen and the dip angle of the bedding has an important effect on the strength of the specimen. When the bedding dip angle is 0° and 90°, the strength of the specimen is 127.20 MPa and 104.61 MPa respectively, and the crack penetrates the bedrock, indicating that the failure mode of the specimen is controlled by the bedrock. When the dip angle of bedding is 30° and 60°, the strength of the specimen is 50.60 MPa and 68.82 MPa respectively, and the crack only exists at the position of bedding when the dip angle of bedding is 30°, which indicates that the failure mode of the specimen is completely controlled by bedding. However, when the dip angle of bedding is 60°, the cracks occur in the bedding and local bedrock and connect through the whole specimen, which indicates that the failure mode of the specimen is determined by both bedding and bedrock. Samples with different bedding inclination angles show different properties after peak. Samples with 30° bedding inclination Angle show better brittleness after peak, while the curves of samples with 0°, 60° and 90° bedding inclination Angle decline slowly after peak, showing greater ductility, as shown in Fig. 4.

## Numerical simulation test

**Figure 3:**
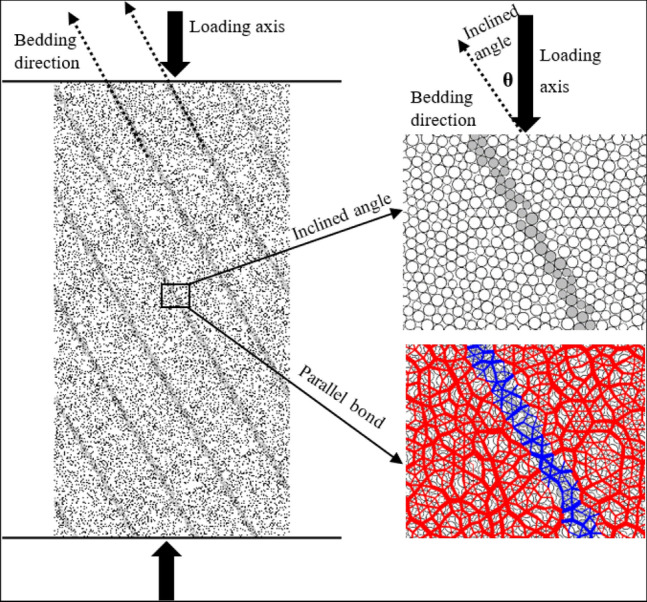
Schematic diagram of numerical model with bedding dip angle of 30°

According to the results of rock mechanics test, the same size specimen, 50 mm in diameter and 100 mm in height, is generated in PFC. The results show that the parallel cementation model can better reflect the mechanical properties of rock material^16,17^. So the linear contact model is adopted between the wall and the particles, and the linear parallel cementation contact model is adopted between the particles. The maximum radius of the particles is 0.45 mm, the minimum radius is 0.3 mm, and the particle diameter ratio is 2:3, the particle density is 2505.9 kg/m^3^ and the damping coefficient is 0.7. Based on the above parameters, taking the bedding dip angle of 90°as an example, there are 11,269 particles, 244 linear contacts and 27,374 linear parallel cementation contacts. The spacing between bedding and bedding is 12 mm.The low strain rate controlled loading method is adopted, and the loading strain rate is 1 × 10^–4^ s^−1^. Figure 3 shows the specimen with bedding dip angle of 30°.

### Meso-parameter calibration

**Figure 4:**
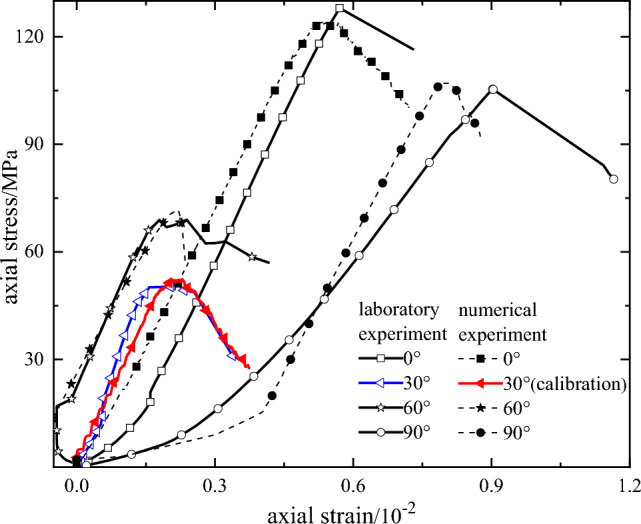
Comparison of the results of rock mechanics experiment and numerical simulation test.

Firstly, the numerical model sample of red shale with a dip angle of 30° is established, and the PB contact model is used in both bedrock and bedding. According to the results of rock mechanics test, the meso-parameters are calibrated by trial and error method. The process is as follows: the model is grouped (bedrock and bedding), the contact between particles is assigned to PB model, the initial value of contact modulus of bedrock and bedding is given first, and the modulus is tested repeatedly, so that the elastic modulus is consistent with the laboratory test. Keep the elastic modulus constant and adjust the stiffness ratio in order to obtain the Poisson's ratio which is consistent with the laboratory test. Finally, the internal friction angle and tension–compression ratio are adjusted so that the peak strength of the numerical test is consistent with that of the laboratory test. The meso-parameters of red shale are shown in Table 2, and the calibration results are shown in in Fig. 4.

**Table 2:** Mesoscopic parameters of numerical model.

Category	Linear part		Cemented part				
	Elastic modulus /GPa	Stiffness ratio	Cementation modulus /GPa	Stiffness ratio	Shear bond strength /MPa	Bonding normal strength /MPa	Internal friction angle /Φ
Bedrock	18	1.5	18	1.5	38	49	45
Bedding	10.8	1.5	10.8	1.5	16	21	32

In order to verify the rationality of the above-mentioned parameters, the numerical models with bedding dip angles of 0°, 60° and 90° are established by using the parameters in Table 2, and the triaxial numerical experiments are carried out. The simulation results are compared with the laboratory test results, as shown in Fig. 4, and the failure modes are shown in Table 3.

**Table 3:** Comparison of failure modes of rock mechanics experiment and numerical simulation test.

Bedding dip angle	0°	30°	60	90°
Laboratory test				
Numerical simulation				
Lei T^21^ Numerical result				
Bona Park^22^ Numerical result				

The peak strength of the bedding dip angle 30°and 90° model is basically consistent with the laboratory test. The peak strength of the numerical test is slightly smaller when the bedding dip angle is 0°, and the error is 2.36%. When the bedding dip angle is 60°, the peak strength of the numerical test is slightly larger, and the error is 3%. The elastic modulus of the numerical test of 0°, 30°and 60° bedding dip angle is basically consistent with that of the laboratory test, and there is a certain deviation in the elastic modulus when the bedding dip angle is 90°, but the stress–strain curve of the numerical test is basically consistent with that of the laboratory test, and the error is also within the controllable range. The stress–strain curves and failure modes of the numerical model are in good agreement with the experimental results, indicating that the meso-parameters are reasonable.

Due to the limitation of field conditions, only four groups of triaxial tests of bedding dip angles of 0°, 30°, 60°and 90°were completed in laboratory tests. In order to deeply analyze the anisotropic mechanical behavior and fracture evolution characteristics of red shale, seven groups of red shale numerical models including 90°, 75°, 60°, 45°, 30°, 15°and 0°were established based on the meso-parameters calibrated above to carry out triaxial compression numerical tests. The confining pressure is consistent with the laboratory test and set to 2 MPa.

### Numerical test results

#### Failure mode

By analyzing the sample failure block diagram and bond failure comparison of samples with different bedding dip angle, the influence of different bedding dip angle on rock failure mode can be clearly understood.

**Figure 5:**
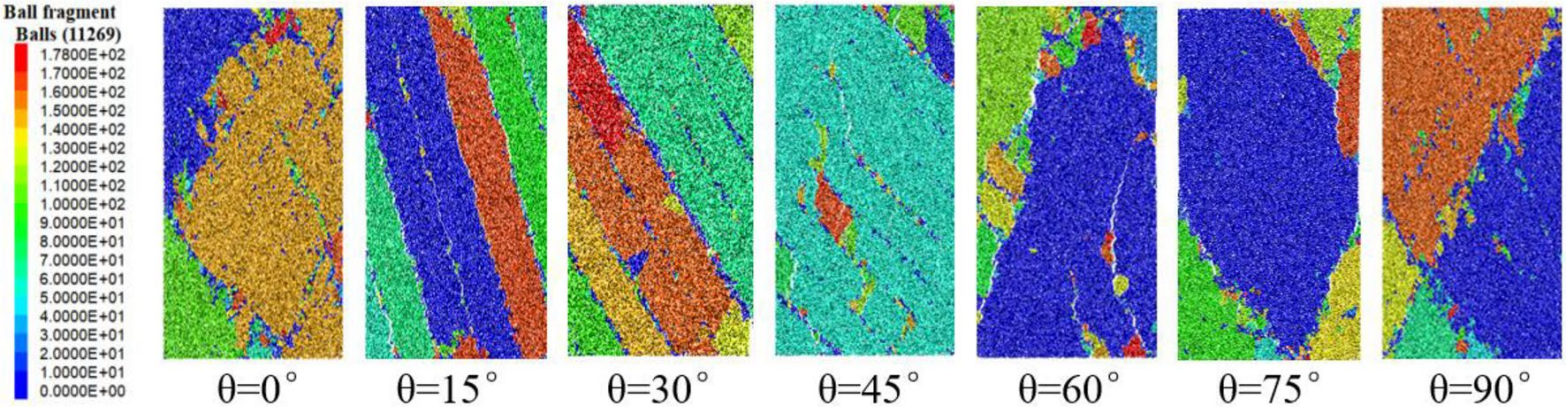
Sample failure block diagram.

In macroscopic view, the failure of layered rock is mainly characterized by two kinds of failure, one is the failure caused by a large number of fractures in the bedrock through the bedding, the other is the failure caused by sliding along the cracks produced by the bedding. When the bedding dip angle is 0°, the rock produces a crack at a certain angle from the loading direction, and the two cracks meet in the middle of the rock, resulting in the failure of the rock. When the bedding dip angle is 15°, 30°and 45°, the rock first breaks in the bedding and produces a slip surface, which leads to rock failure. If the loading continues, the rock will produce cracks in the bedrock between the two adjacent bedding. This process corresponds to the residual strength stage on the stress–strain curve. When the dip angle of bedding is 60°, 75°and 90°, the failure state of rock is similar to that of 0°, and the rock failure is caused by crack penetrating bedding. In particular, when the bedding dip angle is 90°, the specimen forms a conjugate "X" crack, as shown in Fig. 5.

**Figure 6:**
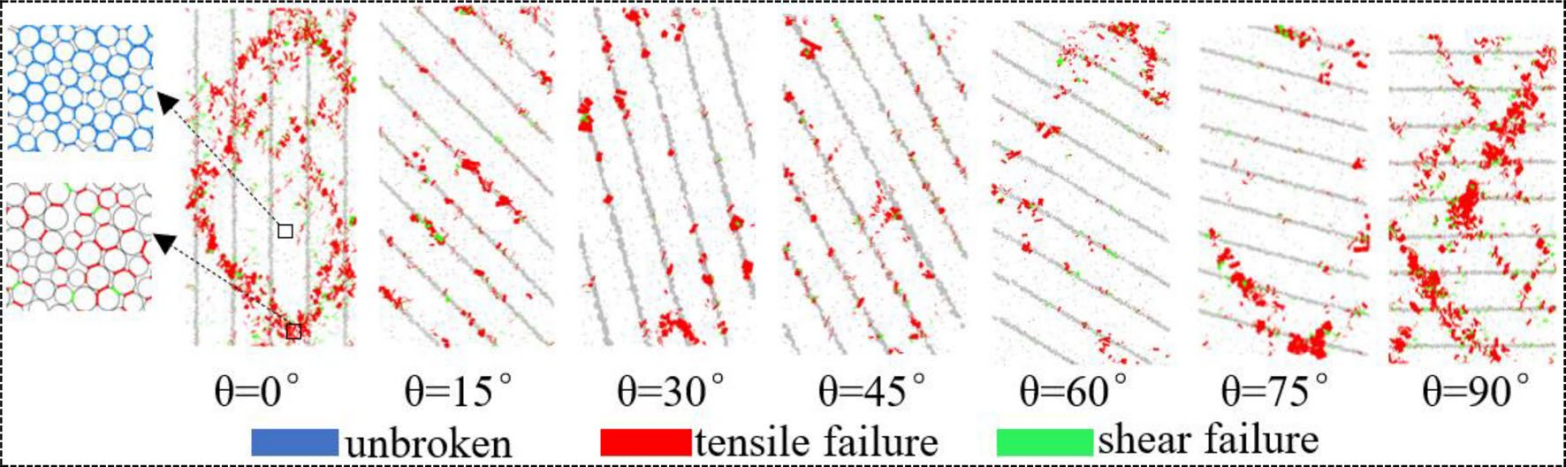
Bond failure comparison.

In meso-view, the essence of rock failure is the fracture of cement bond between particles, and the relative slip of particles, which is shown as the crack during rock failure macroscopically. As shown in Fig. 6, when the bedding dip angles are 0°, 60°, 75°and 90°, most of the cement bond failure occurs in the bedrock and runs through the whole rock sample, resulting in the formation of slip surface and rock failure. When the dip angle of bedding is 15°, 30°and 45°, the failure of cement bond mainly occurs in the position of bedding, which connects the whole bedding to form the failure slip surface, which leads to rock failure. During the numerical simulation test, the failure of bond is mainly tensile failure, accompanied by a small amount of shear failure.

#### Characteristics of fracture evolution

The damage and failure of rock is the macroscopic manifestation of crack initiation, propagation and penetration. PFC can trace the evolution of micro-cracks in the process of rock sample failure, when the bond between particles breaks, it can produce short lines to indicate the crack, and can update the crack location in real time. As shown in Table 4, when the bedding dip angle is 0°, the microcracks occur in the position of the loading plate and in the rock bedding, and a small amount of cracks occur in the bedrock. With the continuation of loading, the micro-cracks in the bedrock gradually expand along a certain angle and connect with the cracks in the bedding, and finally run through the whole specimen, resulting in rock failure. When the bedding dip angle is 15°, the tensile microcrack is formed in the bedrock due to the tension, while the shear microcrack is formed in the bedding due to the shear force. With the continuation of the loading, the tensile crack begins to appear in the bedding, and intersects with the shear crack and runs through the whole specimen, forming a main crack along the bedding, which leads to the rock failure along the bedding. When the bedding dip angle is 30°and 45°, the failure condition is the same as that of 15°, and the cracks are mostly distributed in the bedding. When the bedding dip angle is 60°, at the initial stage of loading, a small number of microcracks will be initiated and connected to form intermittent fracture zones, which are not connected along the bedding. In the later stage of loading, the microcracks initiated in the bedrock propagate rapidly and connect with the fracture zone in the bedding, and finally form a main crack that runs through the whole specimen and connects part of the bedding fracture zone. When the dip angle of the bedding is 75°, the micro-cracks begin to appear in the bedding at the initial stage of loading. With the continuation of loading, the micro-cracks in the bedding propagate to the bedrock at a certain angle, forming a fracture zone through the bedrock and the bedding, in the later stage of loading, the fracture zone intersects and forms the main fracture, which leads to the rock failure. Similarly, when the bedding dip angle is 90°, the microcrack first initiates in the bedding, produces a crack zone at both ends of the rock in the middle of loading, and gradually extends to the middle of the specimen, and finally passes through the specimen to form an "X" type conjugate crack.

**Table 4:**
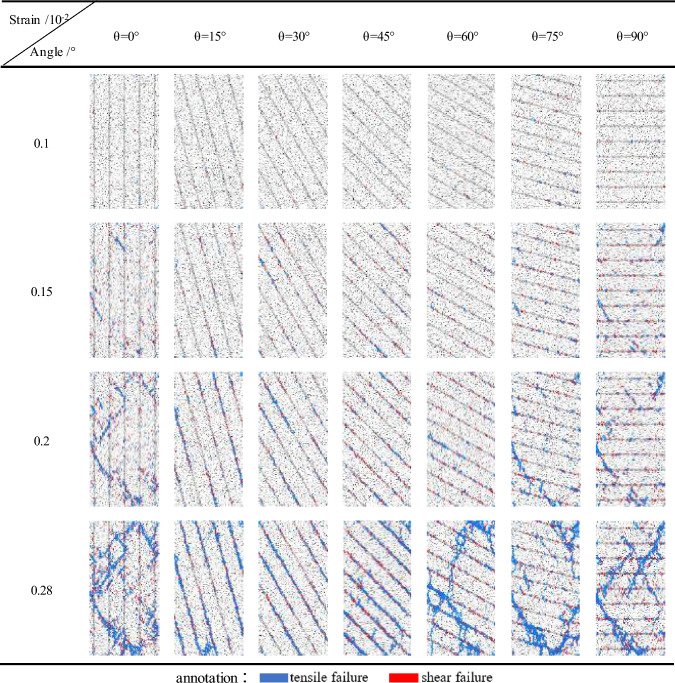
Fracture evolution of samples with different bedding dip angle.

**Figure 7:**
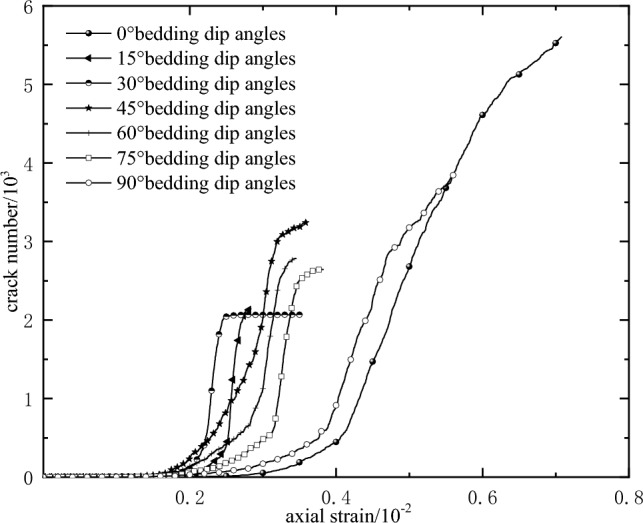
The number of micro-cracks in the process of rock failure.

Figure 7 reflects the evolution of the micro-cracks in the process of rock failure. With the continuous increase of axial strain, the crack shows slow growth stage, accelerated growth stage and tends to stable stage. In the initial stage of loading, micro-cracks gradually appear in the rock, which corresponds to the slow growth stage of the curve. With the continued loading, a large number of micro-cracks are initiated inside, and the macroscopic cracks are formed, which corresponds to the accelerated growth stage of the curve. At the later stage of rock loading, the rock has reached the peak strength, the number of internal microcracks is relatively stable, and the corresponding curve tends to be stable.

**Figure 8:**
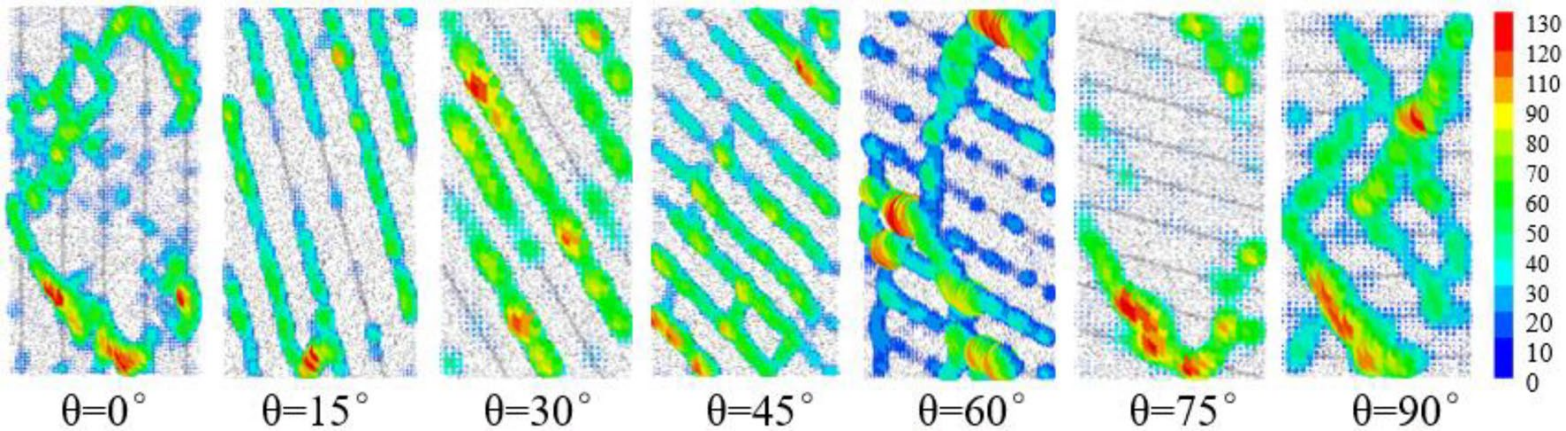
Hot spot diagram of crack distribution in specimens with different bedding dip angles.

The crack evolution diagram can clearly reflect the initiation, propagation and penetration of microcracks in the whole process from loading to failure of rocks with different bedding dip angles, which has intuitive guiding significance for analyzing the development of cracks in rocks with different bedding dip angles. By processing the final state diagram of fracture evolution of red shale with different dip angle of bedding, the qualitative distribution diagram of the number of micro-cracks in different dip angle of bedding rock can be obtained, that is, the hot spot diagram of crack distribution, as shown in Fig. 8. When the dip angle of bedding is 0°, 75° and 90°, a large number of cracks are concentrated near the loading plate, and the cracks are mainly distributed in the bedrock, which indicates that the rock failure is controlled by the bedrock. When the bedding dip angle is 5°, 30°and 45°, a large number of microcracks also appear near the loading plate, but at this time, the cracks are mainly concentrated in the bedding, and there are almost no cracks in the bedrock, indicating that the failure of the rock is controlled by bedding. When the bedding dip angle is 60°, the number of microcracks accounts for almost half of the bedding and bedrock, and a main crack runs through the rock during failure, and part of the bedding fracture zone is connected with the main fissure. It shows that the rock failure is controlled by both bedrock and bedding, and θ = 60° is the turning point of rock failure from bedding control to bedrock control.

**Figure 9:**
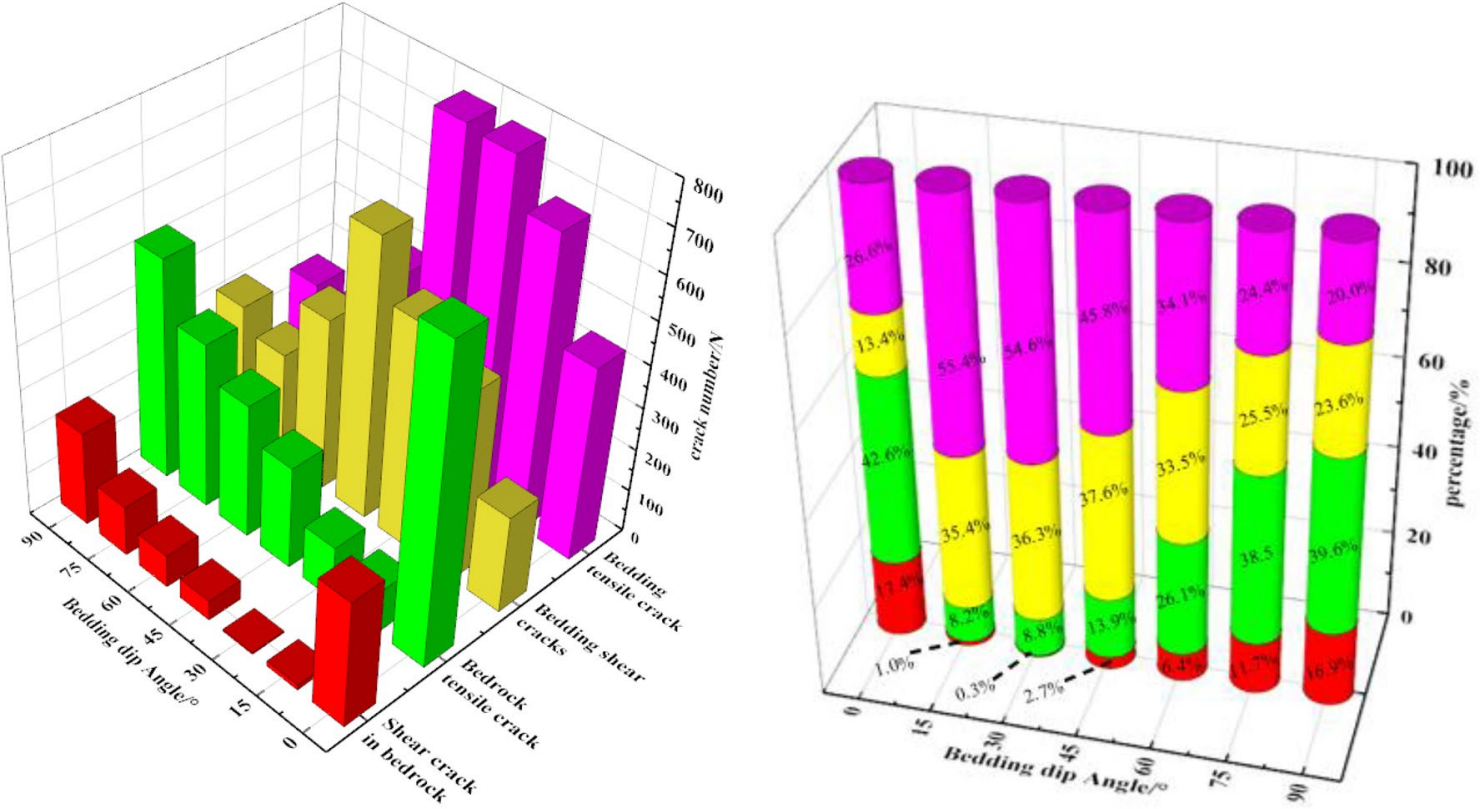
Distribution of cracks in bedrock and bedding with different bedding dip angles.

In order to further analyze the evolution law of cracks with different bedding dip angles, the total number of cracks, the types of cracks and the number of cracks distributed in bedding or bedrock at peak strength are calculated, so as to quantitatively analyze the distribution of microcracks in the initial stage of rock failure, as shown in Fig. 9.

It can be seen from Fig. 9 that there are great differences in the location and number of cracks in rocks with different bedding dip angles. When the rock reaches the peak strength, the total number of tensile cracks is much larger than the number of shear cracks, indicating that the failure of rock is mainly tensile failure, and shear failure occurs in the local part of the rock. When the dip angle of bedding is 15°, 30°, 45°, the microcracks are mainly distributed in the bedding. However, when the dip angle is 0°, 75°, 90°, the microcracks are mainly distributed in the bedrock, and when the dip angle is 60°, the number of microcracks in the bedding is slightly larger than that in the bedrock.

By comparing and analyzing the location and quantity of cracks in red shale with different bedding dip angles, the shear cracks in bedrock reach the minimum value when the bedding dip angles are 15°, 30° and 45°, which account for 1.0%, 0.3% and 2.7% of the total number of cracks respectively. The tensile cracks of bedrock also reach the minimum value when the dip angle of bedding is 15°, 30° and 45°, which account for 8.2%, 8.8% and 13.9% of the total number of cracks respectively. In contrast, the shear cracks and tensile cracks in bedding are close to the maximum, accounting for 90.8%, 90.9% and 83.4% of the total cracks, respectively, indicating that bedding controls the failure mode of rock at this time. When the dip angle of bedding is 0°, 75°and 90°, the shear crack and tensile crack in bedding decrease to the minimum, while the proportion of shear crack and tensile crack in bedrock increases. At the same time, the proportion of tensile crack in bedrock is larger than that in bedding, which indicates that the failure of rock is mainly caused by microcracks initiated in bedrock, that is, the failure mode of rock is controlled by bedrock. When the bedding dip angle is 60°, there is little difference between the tensile crack in the bedrock and the tensile crack in the bedding, which leads to the fracture zone in the bedding and runs through the cracks in the rock.

#### Analysis of rock fabric change

**Figure 10:**
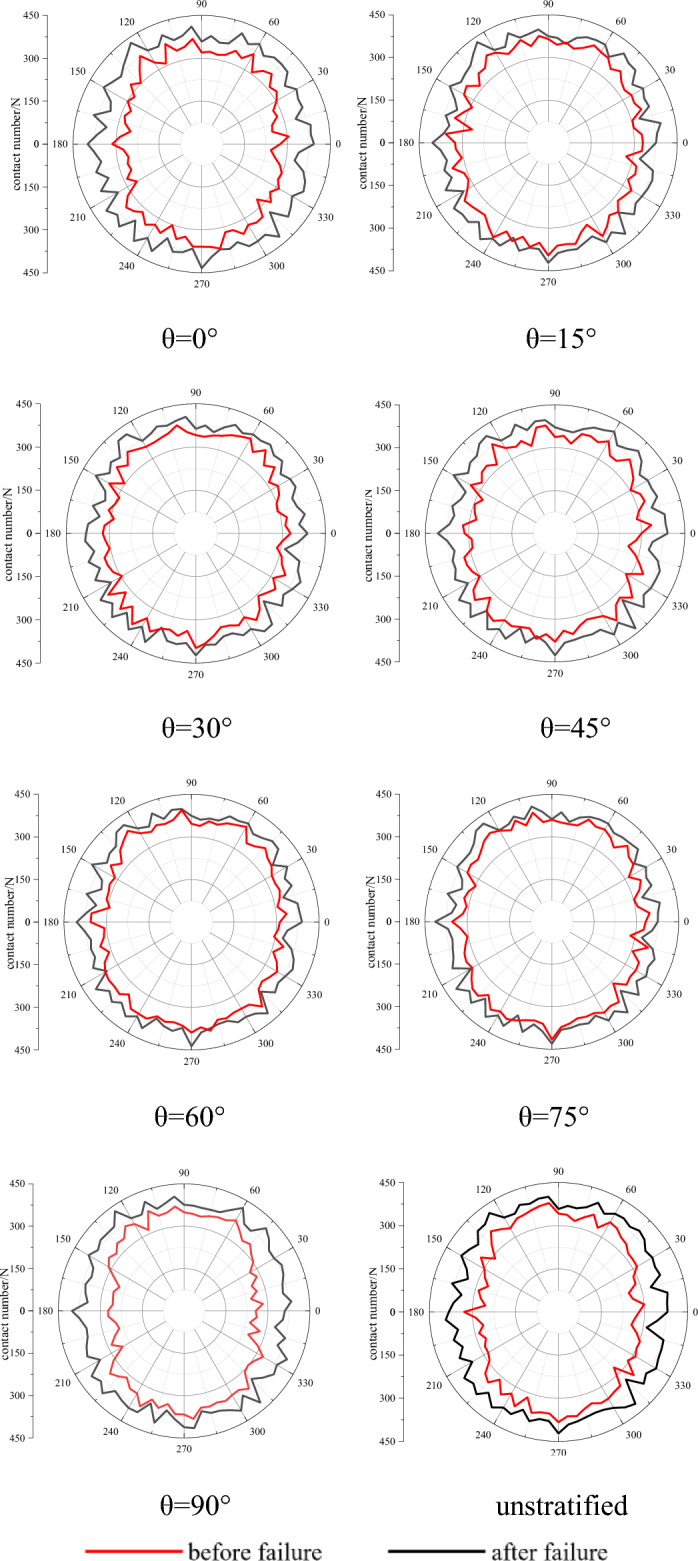
Texture change of specimens with different bedding dip angle before and after loading.

The anisotropy of rock generally shows that the physical–mechanical properties of rock change with the direction. Macroscopically, the rocks of bedding, schist or rhyolitic structure have obvious anisotropy, and microscopically, the strength of anisotropy can be expressed by counting the number and location of primary fractures in rocks. The variation curve of indirect contact number of particles with direction after preloading 2 MPa confining pressure and failure is shown in Fig. 10. The radial direction indicates the number of contacts before and after failure, and the circumferential direction indicates the direction of contact distribution. Before loading, the number of contacts in each direction of the sample is basically the same, that is, the anisotropy of the sample is not obvious before loading. After failure, the contact number in vertical direction is larger than that in horizontal direction, which shows obvious anisotropy. It is worth noting that the difference between the vertical and horizontal directions of the contact numbers of the specimens without bedding and the dip angles of 0°, 90° of bedding is greater than that of 15°, 30°, 45°, 60°, 75° of the dip angles of bedding, that is, the greater the difference is, the flatter the curve.

## Results and discussion

### Bedding effect of red shale

**Figure 11:**
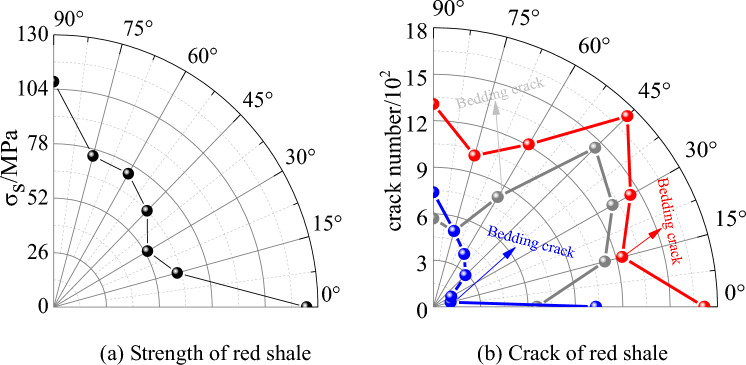
The variation of strength and crack of red shale with bedding dip angle.

Bedding is regarded as the primary defect of rock, and different bedding dip angles have great influence on rock strength. As shown in Fig. 11a, when the bedding dip angle is 30°, the peak strength is the minimum, while when the bedding dip angle is 0°, the strength value is about 2 times that of 30°, indicating that the bedding dip angle is an important factor controlling the strength of red shale. Figure 11b shows the variation of cracks in bedding, cracks in bedrock and total cracks with bedding dip angle when the specimen reaches peak strength. When the bedding dip angle is 30° ~ 45°, there is almost no crack in the bedrock, and the failure of red shale is controlled by the crack in bedding. In the case of other bedding dip angles, the number of cracks in the bedding is slightly higher than that in the bedrock, because the bedding belongs to the weak structural plane, and microcracks always appear first when loading. however, the difference of the dip angle determines whether the crack in the bedding controls the final failure mode of the specimen.

### Evolution mechanism of particle displacement field

**Figure 12:**
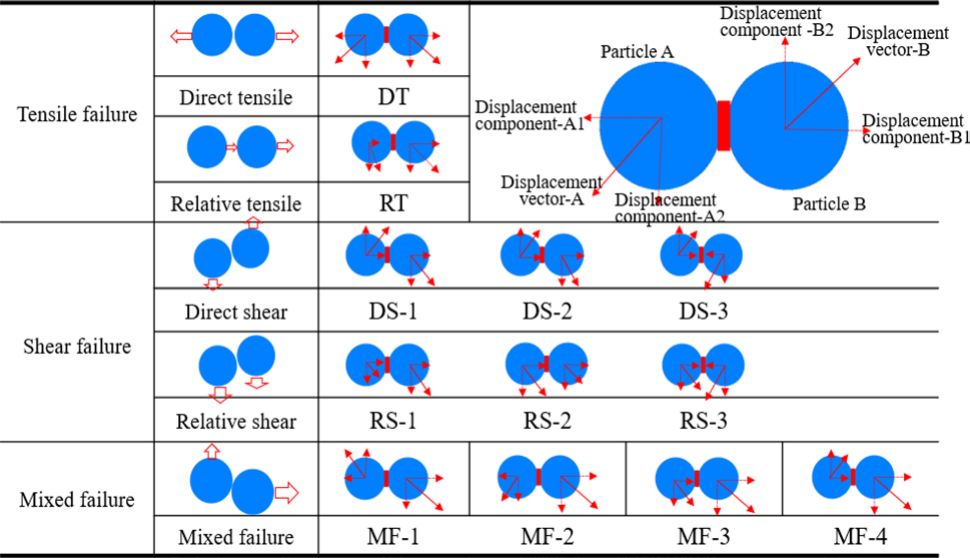
Schematic diagram of particle displacement under different crack modes.

In the above, the crack evolution of samples with different bedding dip angles during failure has been studied macroscopically, including crack development process, crack distribution location and number, crack type. Microcrack is a microscopic manifestation of relative slip caused by the fracture of chemical bonds between particles. when the force acting on the particles exceeds the fracture strength of the particles, the chemical bonds will break. Through the analysis of the particle displacement field during the failure of the specimen, the microscopic evolution mechanism of the crack can be further understood. Figure 12 is a schematic diagram of particle displacement under different crack modes summarized by predecessors. A detailed explanation can be found in reference^23–26^.

**Figure 13:**
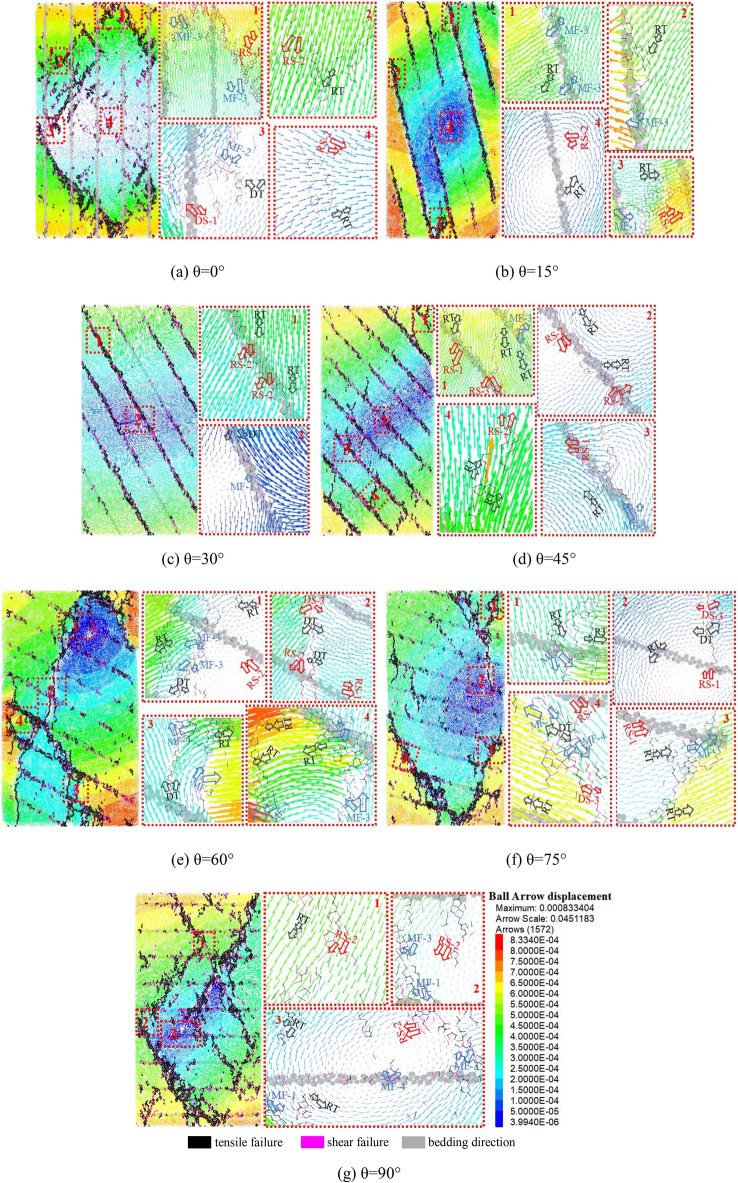
Displacement field driven by crack with different dip angle.

Figure 13 shows the crack initiation mode of different bedding dip displacement, the black hollow arrow indicates the tensile failure mode, the red hollow arrow indicates the shear failure mode, and the blue hollow arrow indicates the mixed failure mode. The black line indicates tensile failure, the magenta lines indicate shear failure and gray indicates bedding direction.

When the bedding dip angle is 0°, the displacement field dominated by mixed failure MF-3 often occurs near the loading plate, tensile failure or shear failure occurs in the process of crack propagation, and the annular displacement field induced by mixed failure MF-2 occurs when the cracks converge. As shown in Fig. 13a, the mixed failure mode (MF-2) dominates and the annular displacement field driven by tensile failure mode (DT)and shear failure mode (DS-1) occurs during crack propagation.

When the bedding dip angle increases to 15°, an annular displacement field is formed in the center of the sample, and shear failure (RS-2) and tensile failure (RT) occur at the bedding position. Away from the center of the specimen, due to the large force, mixed failure (MF-3) and relative tensile failure (RT) occur when the displacement is transferred to the bedding position. After the displacement is transferred through the bedding, the displacement field is driven by the crack, as shown in Fig. 13b. When the specimen is close to failure, mixed failure (MF-1), shear failure (RS-1) and tensile (RT) occur along the bedding position in the middle of the loading plate, which further drives the change of the displacement field in the bedrock and causes cracks in the bedrock.

The displacement at the end of the specimen is transmitted by tensile failure (RT, DT) and shear failure (RS-2). The mixed failure (MF-1) is produced at the middle of the specimen due to the different direction and size of the displacement field from the upper and lower end surfaces, promote the development of cracks in the bedding, as shown in Fig. 13c. During the whole process, the change of displacement field is relatively single, but there is no crack in the bedrock, and the crack will be produced when the bedding with weak lithology is encountered, which leads to the failure of the rock along the bedding completely, the peak strength of the specimen comes entirely from the failure of bedding, which is the reason why the peak strength at 30° dip angle is the smallest.

The propagation of some cracks in the bedrock is magnified and compared when the bedding dip angle is 45°, as shown in Fig. 13d. During the failure of the sample, the main crack is formed in the bedding position, and the associated crack occurs in part of the bedrock. In the early stage of loading, the displacement field of the specimen accelerates the crack to form and propagate into the main crack. When the specimen is loaded to the middle stage, that is, when the specimen is about to reach the peak strength, the displacement transmission changes when the specimen is driven by the bedding fracture, and the crack occurs in the bedrock, the relative tensile failure (RT), shear failure (RS-1) and shear failure (RS-2) types occur in the bedrock.

Figure 13e is the displacement field with a bedding dip of 60°. Driven by tensile failure(RT, DT), shear failure (RS-2) and mixed failure (MF-3), the displacement field develops into a circular displacement field, at the same time promote the development of crack. Driven by the relative tensile failure (RT) and the mixed failure (MF-3) modes, the displacement field evolves gradually to a circular displacement field with a small area as the center. The displacement field is similar to that of 60° when bedding dip angle is 75°.

When the dip angle of bedding is 90°, the main crack through the specimen occurs when the specimen is damaged, shown in Fig. 13g. It is found that the displacement field at the turning point of the crack is different in magnitude and direction: the displacement field on the left is slightly to the left driven by the crack, when the strength of the chemical bond between particles is less than the tensile or shear force produced by the displacement field, the fracture of the chemical bond results in the crack turning. Driven by shear failure (RS-2) and mixed failure (MF-3, MF-1), the displacement field propagates to the center of the specimen and forms a circular displacement field as shown in Fig. 13g.

The above analysis shows that the type of displacement field varies with the dip angle of bedding. When the bedding dip angle is 15°, 30°and 45°, the displacement fields of most specimens are tensile failure (DT and RT) and shear failure (DS and RS). When the bedding dip angles are 0°, 60°, 75°and 90°, the displacement field is usually dominated by mixed failure modes (MF-1, MF-2 and MF-3). Driven by the mixed failure mode cracks, the annular displacement fields of relative tensile failure (RT) and shear failure (RS-1) are produced.

### On-site reference significance

The experimental study shows that the strength of red shale is the lowest when the bedding dip angle is 30°, and the shear failure occurs most easily in the bedding. Furthermore, the bedding angle under the laboratory scale is transformed into the tendency of the bedding plane under the engineering scale, and the critical threshold of the angle between the roadway strike and the stratified plane is obtained, so as to guide the site to consciously avoid the threshold and reduce the influence of surrounding rock bedding on roadway failure.

## Conclusions

1. The numerical simulation test shows that the failure mode of red shale is controlled by bedrock when the bedding dip angle is 0° and 60° ~ 90°. When the bedding dip angle is 15° ~ 45°, the rock failure mode is controlled by bedding. The compressive strength of rock is the minimum when the bedding dip angle is 30°and the maximum at 0°, which is about 2 times of the minimum.
2. In the failure process of red shale, the cracks with different bedding dip angles show slow growth stage, accelerated growth stage and stable stage with axial strain. The whole failure process is dominated by tensile cracks, accompanied by a few shear cracks.
3. The type of displacement field varies with the bedding dip angle: The bedding dip angle is 15° ~ 45°, most displacement field types are tensile failure (DT and RT) and shear failure (DS and RS). When the bedding dip angle is 60° ~ 90° and θ = 0°, the displacement field type is usually dominated by mixed failure modes (MF-1, MF-2 and MF-3).

## Data Availability

The data that support the findings of this study are available from the corresponding author upon reasonable request.
