# Author Correction: Experimental study on failure mode and fracture evolution characteristics of red shale in Kaiyang Phosphorus mining area

**DOI:** 10.1038/s41598-024-74028-w

**Published:** 2024-10-04

**Authors:** Zhenqian Ma, Lang Zhou, Shaojie Zuo, Jimin Zhang

**Affiliations:** School of Mining, Guizhou University, Guiyang, 550025 Guizhou Province China

Correction to: *Scientific Reports *10.1038/s41598-024-60981-z, published online 03 May 2024

The original version of this Article contained an error in Reference 13, which was incorrectly given as:

Yang, S. Q., Sun, B. W. & Tian, W. L. Numerical modeling of jointed rock samples under unconfined and confined conditions to study peak strength and failure mode. *Arab. J. Geosci.***14**, 174 (2021).

The correct reference is listed below:

As’habi, F. & Lakirouhani, A. Numerical modeling of jointed rock samples under unconfined and confined conditions to study peak strength and failure mode. *Arab. J. Geosci.***14**, 174. 10.1007/s12517-021-06569-7 (2021)

The original Article has been corrected.

